# Stability Indicating RP-HPLC Method for Simultaneous Determination of Simvastatin and Ezetimibe from Tablet Dosage Form

**DOI:** 10.4103/0250-474X.65028

**Published:** 2010

**Authors:** R. P. Dixit, C. R. Barhate, S. G. Padhye, C. L. Viswanathan, M. S. Nagarsenker

**Affiliations:** Departments of Pharmaceutics, Bombay College of Pharmacy, Kalina, Santacruz (E), Mumbai-400 098, India; 1 Department of Pharmaceutical Chemistry, Bombay College of Pharmacy, Kalina, Santacruz (E), Mumbai-400 098, India

**Keywords:** Ezetimibe, reverse phase high performance liquid chromatography, simvastatin, stress degradation, tablets

## Abstract

A simple, specific and sensitive reverse phase high performance liquid chromatographic method was developed and validated for simultaneous determination of ezetimibe and simvastatin from pharmaceutical dosage forms. The method uses C18 ODS Hypersil column and isocratic elution. The mobile phase composed of acetonitrile:phosphate buffer (pH 4.5, 0.01M) in the ratio of 65:35 v/v was used at a flow rate of 1.0 ml /min. UV detector was programmed at 232 nm for first 10 min and at 238 nm for 10 to 20 min. All the validation parameters were in acceptable range. The developed method was effectively applied to quantitate amount of ezetimibe and simvastatin from tablets. The method was also applied suitably for determining the degradation products of ezetimibe and simvastatin.

Simvastatin (SMV) is -(+)-{1*S*,3*R*,7*S*,8*S*,8*aR*)-1,2,3,7,8,8*a*-hexahydro-3,7-dimethyl-8-[2-(2*R*,4*R*)-tetrahydro-4-hydroxy-6-oxo-2H-pyran-2-yl]-I-naphthyl-2,2-dimethyl butanoate (fig. 1). It acts by inhibiting HMG CoA reductase and is used for the treatment of hypercholesterolemia. After oral administration, this prodrug is converted into β hydroxy acid of simvastatin, which is a potent inhibitor of HMG CoA reductase, a key enzyme required for the synthesis of cholesterol in liver[1].

**Fig. 1:**
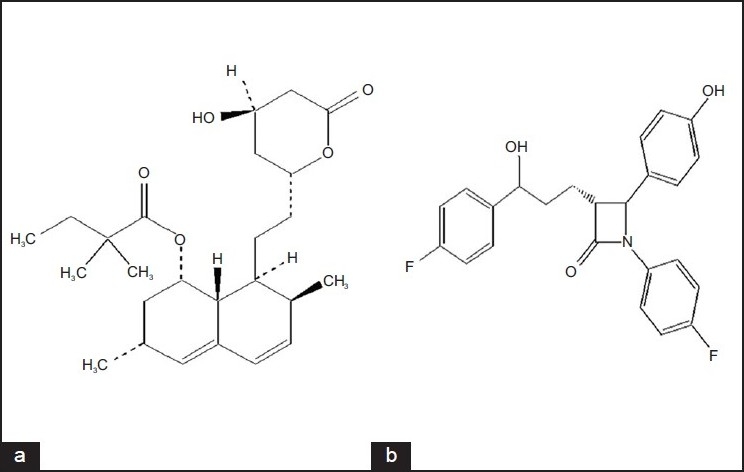
Chemical structures of drug molecules (a) SMV is simvastatin and (b) EZE is ezetimibe

Ezetimibe (EZE) is [(3*R*,4*S*)-1-(4-fluorophenyl)-3-[(3*S*)-3-(4-fluorophenyl)-3-hydroxypropyl]-4-(4-hydroxyphenyl)-2-azetidinone] (fig. 1). It is a selective cholesterol absorption inhibitor used in the treatment of primary hypercholesterolemia. It inhibits the absorption of biliary and dietary cholesterol from small intestine without affecting absorption of fat soluble vitamins, triglycerides and bile acids. After oral administration, EZE is metabolized into its glucuronide in the liver and small intestine, which is also active in prevention of absorption of cholesterol. EZE does not have significant pharmacokinetic interactions with other lipid lowering drugs as it does not influence the activity of cyotochrome P450. EZE is administered at the dose of 10 mg with and without SMV[2]. Both the drugs are marketed as a combination therapy in USA as a trade name Vytorin^TM^ for treatment of hyperlipidemia.

SMV alone can be estimated by various methods reported in the literature such as high performance liquid chromatography (HPLC) with UV detection[3], liquid chromatography coupled with tandem mass spectroscopy[4], UV spectrophotometry[5]. HPLC method for determination of EZE from pharmaceutical dosage form has been reported in the literature[6]. Reports are available which describe a stability indicating method for determination of SMV as well as for EZE with its degradation products and impurities[7,8]. Simultaneous determination of SMV and EZE from pharmaceutical dosage form by dual mode gradient liquid chromatography was also reported[9]. The method involved use of C8 column and elution was accomplished by the application of a dual-mode solvent and flow-rate gradient system. Detection was carried out using a diode-array detector set at 240 nm. A Stability indicating HPTLC method for simultaneous estimation of SMV and EZE was also reported[10].

The present work is aimed at development of a sensitive, specific and validated reverse phase high performance liquid chromatographic method for simultaneous determination of SMV and EZE from the dosage form and its degradation products formed under stress degradation of both SMV and EZE.

## MATERIALS AND METHODS

Simvastatin was donated by Sun Pharmaceuticals Ltd., Mumbai, India. Ezetimibe was generously gifted by Lupin Pharmaceutical Ltd., Pune, India. Acetonitrile (HPLC grade) was purchased from Qualigens fine chemicals, Mumbai, India. Distilled, 0.45 µm filtered water used for HPLC analysis and preparation of buffer. Buffers and all other chemicals were analytical grade. The chromatographic system consisted of the following components all from Jasco corporation (Tokyo, Japan): A UV/Vis detector (UV 2075 plus) covering the range of 200-400 nm and interfaced to a computer for data acquisition and a recorder model Star 800 interface module. A PU 2080 plus solvent delivery system, a Rheodyne, 50 µl loop injector. An ODS Hypersil column (250×4.6 mm, 5 µm) was used (Thermo Electron Corporation, USA) and mixture of acetonitrile and phosphate buffer (pH 4.5, 0.01M) in the proportion of 65:35 v/v was used as mobile phase at 1.0 ml/min. The column was maintained at ambient temperature. Detector was programmed at 232 nm for detection of EZE for 10 min and 238 nm for detection of SMV from 10 min to 20 min.

### Stock solution and calibration standard sample:

A stock solution was prepared by dissolving accurately weighed 25 mg of SMV and EZE in a 25 ml volumetric flask to obtain 1 mg/ml solution using HPLC grade methanol. The stock solution was diluted suitably with methanol to obtain concentrations in the range of 2 to 10 µg/ml and calibration curves were plotted.

### System suitability:

The system suitability was assessed using five replicate analyses of drugs at concentration of 2 µg/ml. The acceptance criterion was ±2 % of coefficient of variation (% CV) for retention times and peak areas for both drugs.

### Detection and quantitation limits:

Limit of detection (LOD) and limit of quantitation (LOQ) were obtained from signal to noise ratio. The detection limit was defined as the lowest concentration level resulting in a peak area of three times the baseline noise. The quantitation limit was defined as the lowest concentration level that provided a peak area with a signal-to-noise ratio higher than 10.

### Accuracy and precision:

Accuracy of the assay method was determined for both intra-day and inter-day variations using the triplicate analysis of drugs at three concentrations viz. 2 µg/ml, 6 µg/ml and 10 µg/ml. These samples are denoted as QC samples. Precision of the assay was determined by repeatability (intra-day) and intermediate precision (interday) in triplicate. Repeatability refers to the use of the analytical procedure over a short period of time that was evaluated by assaying the QC samples during the same day. Intermediate precision was assessed by comparing the assays on different days (3 days).

### Specificity:

Specificity of the method was determined by subjecting the sample solution (5 µg/ml) to degradation after addition of pH 7.4 buffer in order to verify that none of the degradation products interfered with the quantitation of drug.

### Stress degradation studies:

Stress testing of the drug substance can help identify the likely degradation products, stability of the molecule and also validate the stability and specificity of the analytical procedures. For degradation studies, solutions of drugs in methanol containing 1000 µg/ml were mixed with 0.1N HCl, pH 7.4 phosphate buffer and 10% H_2_O_2_ to obtain the concentration of drug 5 µg/ml. The solution mixed with H_2_O_2_ was heated at 60° for 30 min, solution mixed with 0.1N HCl and phosphate buffer pH 7.4 solutions were kept at room temperature for degradation to occur. Fifty microlitres of the solutions were injected in HPLC and analyzed.

### Application of method to dosage form:

The developed and validated HPLC method was applied for determination of SMV and EZE from dosage forms. SMV and EZE tablets of 10 mg strength from Ranbaxy Labs (Simvotin^TM^ EZ 10) were evaluated for SMV and EZE content in tablets. The tablets were powdered and powder equivalent to 10 mg of drugs was weighed. The weighed samples were placed in extraction flask and methanol was added to extract the drug. The suspension was sonicated for 10 min and stirred using a CM 101 cyclomixer (Remi Equipments, Mumbai, India). The mixture was centrifuged using Minispin centrifuge (Eppendorf, Germany) for 10 min at 10, 000 rpm. The supernatant was diluted suitably to obtain 10 µg/ml concentration of both drugs. The solutions were injected into HPLC and analyzed for drug content.

## RESULTS AND DISCUSSION

**Fig. 2:**
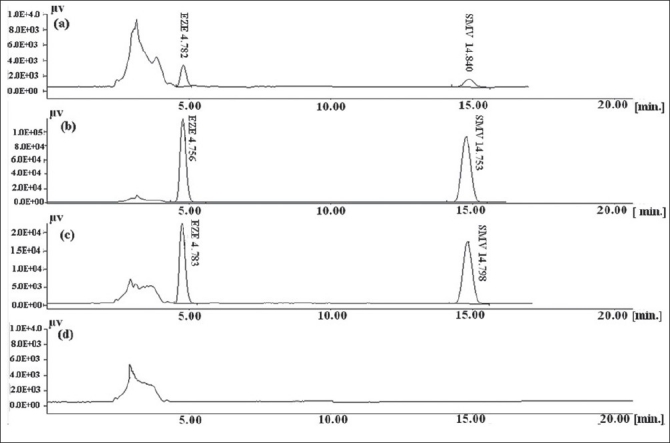
Chromatograms of SMV and EZE (a) LOQ of EZE and SMV, (b) standard solution (10 µg/ml), (c), Standard solution (2 µg/ml). EZE and SMV elute approximately at 4.7 min and 14.7 min. (d) Methanol devoid of drugs. SMV is simvastatin and EZE is ezetimibe

Both SMV and EZE have limited aqueous solubilities hence methanol was used for the extraction of drugs from the formulations and for preparation of stock solutions. EZE and SMV have λ_max_ at 232 nm and 238 nm respectively, so HPLC analysis was done at 232 nm for EZE and 238 nm for SMV. Mobile phase optimization was initiated using ACN and phosphate buffer pH 4.5 at the flow rate of 1.5 ml/min[11]. The flow rate was decreased from 1.5 ml/min to 1.0 ml/min to resolve the degradation product from main drug peaks. The peak shape and separation was found to be good when a mobile phase composition of 65:35 (v/v, acetonitrile:phosphate buffer pH 4.5) was used at the flow rate of 1.0 ml/min. Although a HPLC gradient method using diode-array detector has been reported for the determination of SMV and EZE from pharmaceutical dosage form, the above developed method has few advantages on the reported method[9]. The method developed above is simple, uses isocratic flow system and UV detector for detection of drug. The chromatograms are given in the fig. 2.

The calibration curve constructed was evaluated by its correlation coefficient. The peak area was linear in the range of 2 to 10 µg/ml (Table 1). The correlation coefficients for both the calibration plots of drugs were more than 0.9995.

**TABLE 1:** REGRESSION ANALYSIS OF LINEARITY DATA OF EZE AND SMV

Parameter	Mean±SD	
	EZE	SMV
Slope	150472.8±1554.7	190994.8±1492.8
Intercept	9668.0±4110	7102.5±4581.9
Correlation coefficient (r^2^)	0.9996±0.0004	0.9998±0.0003

SMV is simvastatin and EZE is ezetimibe, SD is the standard deviation and n=6

The percent CV of retention time for both the drugs was less than 0.3 % indicating high stability of the system. The percent CV of peak area was with in the range of 2 % limit signifying suitability of the system (Table 2). The USP tailing factor was 1.205±0.4 (mean± % CV) for EZE and 1.049±0.3 (mean± % CV) for SMV.

**TABLE 2:** SYSTEM SUITABILITY STUDY

Statistical parameter	EZE (2 µg/ml)			SMV (2 µg/ml)		
	Retention time (min)		Peak area	Retention time (min)		Peak area
Mean±SD	4.69±0.01		285301.80±3567.71	14.52±0.04		368756.20±2720.95
% CV	0.17		1.25	0.26		0.74
LOD		20.0 ng/ml			20.0 ng/ml	
LOQ		100.0 ng/ml			100.0 ng/ml	

SD is the standard deviation; % CV is coeffi cient of variance; LOD, limit of detection and LOQ, limit of quantifi cation; n=5.

Fig. 2 shows the chromatogram of methanol devoid of drugs, standard concentrations of SMV and EZE and LOQ of drugs. The method was inferred to be sensitive as evident from triplicate injections for LOQ (Table 2). The % CV was 1.27 % for EZE and 7.63% for SMV, respectively.

The accuracy and precision calculated for samples during intra day and inter day run are given in Table 3. The intra day accuracy ranged from -2.09 % to 0.18 % and percent coefficient of variation for precision from 0.72 % to 2.80 %. The results obtained from intermediate precision (inter-day) also indicated a good method precision. All the data were in the acceptable range of criteria of 5%.

**TABLE 3:** INTRA- AND INTER-DAY ACCURACY AND PRECISION OF HPLC ASSAY FOR EZE AND SMV

Nominal concentration						
Parameters	EZE			SMV		
	2 µg/ml	6 µg/ml	10 µg/ml	2 µg/ml	6 µg/ml	10 µg/ml
Day 1						
Mean±SD	1.96±0.02	5.93±0.15	10.05±0.11	1.97±0.01	5.98±0.17	10.02±0.10
% CV	1.21	2.49	1.14	0.72	2.80	0.99
% Bias	-2.09	-1.18	-0.33	-1.69	0.5	0.18
Day 2						
Mean±SD	1.71±0.003	6.43±0.23	10.57±0.36	1.64±0.01	6.12±0.22	9.89±0.34
% CV	0.13	3.51	3.40	0.71	3.60	3.43
% Bias	-0.86	7.18	5.73	-1.07	2.01	0.167
Day 3						
Mean±SD	2.03±0.04	6.27±0.05	10.59±0.11	1.95±0.005	6.14±0.05	10.35±0.08
% CV	2.31	0.79	1.00	0.304	0.76	0.75
% Bias	1.65	4.45	5.92	-2.41	2.25	3.54

SD: standard deviation; % CV: coeffi cient of variance; n=3.

**Fig. 3:**
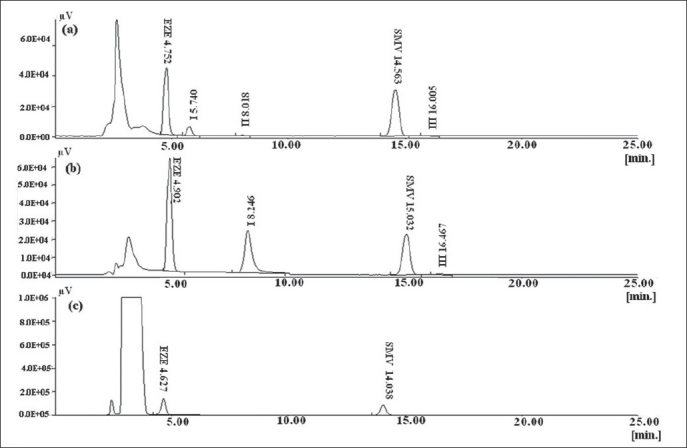
Chromatograms depicting degradation products of EZE and SMV and specifi city of method (a) Degradation pattern EZE and SMV in phosphate buffer pH 7.4 (b) degradation pattern of EZE and SMV in acid, (c) degradation pattern of EZE and SMV in H_2_O_2_. SMV is simvastatin and EZE is ezetimibe

In the chosen chromatographic condition, specificity was indicated by absence of interference at retention times of peaks of interest. The specificity of method is given in fig. 3. The degradation products of both drugs did not interfere with the retention times of both drugs.

The HPLC method developed was also useful for the determination of degradation products formed when the drugs were treated with acid, phosphate buffer pH 7.4 and hydrogen peroxide. The previously reported method was not applied for the separation of degradation products from the parent drugs and no method has been reported for the same[9]. Individual drugs were exposed to acid, phosphate buffer pH 7.4 and H_2_O_2_ to confirm the assignment peaks observed due to degradation of SMV/EZE in the chromatogram of SMV and EZE mixture (data not shown). The stress testing involved effect of heat, oxidation and pH on the stability of drug in solution. When 0.1 N NaOH was used, SMV and EZE were completely degraded in 30 min even when stored at room temperature and no drug peaks were seen. Hence phosphate buffer pH 7.4 was used as a medium for degradation. Duration of 30 min was sufficient to form the degradation products without losing the drug peaks. In this case EZE degradation peak was detected following the peak of EZE, which was distinctly separated from EZE peak (fig. 3). Two peaks of SMV degradants were observed. The major degradation peak of SMV was eluted at 8 min and second degradation peak was eluted at 16 min. The tailing factor of all the peaks was less than 2 (Tables 4 and 5). These degradation peaks were also observed when SMV solution was exposed to acid. No additional peak was observed when EZE was treated with 0.1N HCl. In acidic conditions, EZE remained stable and the content of EZE was in the range of 95% - 105%. In case of oxidative degradation there was a significant decrease in peak area of SMV with no additional peak of degradant, indicating that SMV was degraded to a great extent by oxidation. There was no significant change in peak area of EZE and no degradation product was observed which indicates EZE is stable to oxidative stress.

**Fig. 4:**
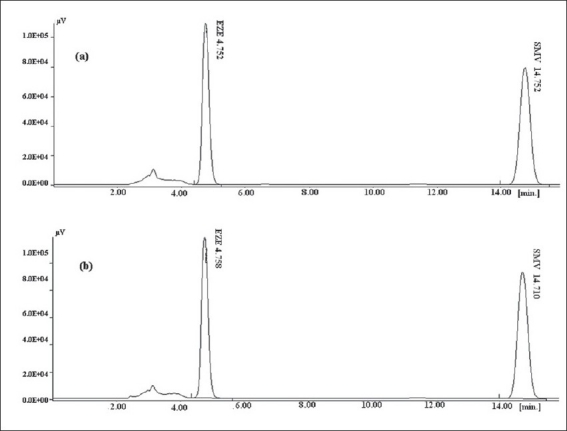
Comparison of chromatogram of marketed formulation with the chromatogram of standard solution. (a) Chromatogram of marketed formulation, (b) chromatogram of standard solution (10 µg/ml)

The method was used for determination of both the drugs from pharmaceutical dosage form. Tablets containing EZE and SMV of Ranbaxy labs (Simvotin^TM^ EZ 10) were evaluated for content of SMV and EZE. The product was analyzed in triplicate (Table 6). None of the tablet ingredients interfered with the analysis of both the drugs as seen in fig. 4.

**TABLE 4:** SPECIFICITY DATA OF EZE

Sample	Retention time (min)	Area	USP tailing
EZE standard (5 µg/ml)	4.755	776822.30	1.126
EZE in pH 7.4 phosphate buffer	4.752	542177.70	1.019
EZE in acidic condition	4.902	792015.12	0.959
EZE in H_2_O_2_	4.627	730859.00	0.886
EZE unknown degradant (in pH 7.4 phosphate buffer)	5.740	71859.48	1.121

The solutions mixed with 0.1N HCl (acidic condition) and phosphate buffer pH 7.4, were kept at room temperature, whereas solution mixed with H_2_O_2_ was heated at 60° for 30 min. EZE denotes ezitimibe.

**TABLE 5:** SPECIFICITY DATA OF SMV

Sample name	Retention time (min)	Area	USP tailing
SMV standard solution (5 µg/ml)	14.73	969566.33	0.996
SMV in pH 7.4 phosphate buffer	14.563	599322.75	0.983
SMV in acidic condition	15.032	457377.32	0.976
SMV in H_2_O_2_	14.038	683473.69	1.034
SMV unknown degradant I (In acidic condition)	8.245	498998.59	1.559
SMV unknown degradant II (In acidic condition)	16.647	12434.00	1.088
SMV unknown degradant I (In pH 7.4 phosphate buffer)	8.018	4899.55	0.912
SMV unknown degradant II (In pH 7.4 phosphate buffer)	16.005	5648.66	1.029

The solutions mixed with 0.1N HCl (acidic condition) and phosphate buffer pH 7.4, were kept at room temperature, whereas solution mixed with H_2_O_2_ was heated at 60° for 30 min. SMV is simvastatin

**TABLE 6:** CONTENT OF EZE AND SMV IN TABLET DOSAGE FORM

% Drug content	EZE	SMV
Mean±SD	100.17±0.416	90.46±0.27
% CV	0.42	0.31

SMV is simvastatin and EZE is ezetimibe, SD: standard deviation; % CV: coeffi cient of variance; n= 3

A rapid, simple and specific reverse phase HPLC method has been developed for simultaneous determination of EZE and SMV from tablet dosage form. The method was validated for accuracy, precision, linearity. Application of this method for simultaneous determination of SMV and EZE from tablet dosage form showed that neither the degradation product nor the excipients interfere with the determination.
